# The orphan nuclear receptor Nr4a1 contributes to interstitial cardiac fibrosis via modulation of cardiac fibroblast and macrophage phenotype

**DOI:** 10.1007/s00018-024-05513-8

**Published:** 2024-12-07

**Authors:** Alexander Widiapradja, Heather Connery, Martyn Bullock, Ainsley O. Kasparian, Roderick Clifton-Bligh, Scott P. Levick

**Affiliations:** 1 Department of Physiology, Pharmacology, and Toxicology, West Virginia University Health Sciences Center, Morgantown, WV USA; 2 Kolling Institute, St Leonards, NSW Australia; 3 The University of Sydney, Camperdown, NSW Australia; 4 Royal North Shore Hospital, St Leonards, NSW Australia

**Keywords:** Inflammation, Cardiac remodeling, Heart failure, Diastolic function

## Abstract

The orphan nuclear receptor Nr4a1 has complex biological functions and has been implicated in numerous diseases, including cardiovascular disease. While protective in atherosclerosis and myocardial ischemia, Nr4a1 has been shown to cause cardiac fibrosis in non-ischemic adverse remodeling of the heart. However, mechanisms underlying these actions are still poorly understood. Accordingly, we sought to: (1) understand the contribution of Nr4a1 to the inflammatory environment including macrophage phenotype; and (2) determine the contribution of Nr4a1 to cardiac fibroblast phenotype in the fibrotic heart. Wild type and Nr4a1^−/−^ mice were infused with angiotensin II (1500 ng/kg/min) to induce cardiac fibrosis and diastolic dysfunction. Nr4a1 deletion prevented cardiac fibrosis and maintained normal diastolic function. We determined that macrophages lacking Nr4a1 had distinctly different phenotypes to wild type macrophages, with Nr4a1 deletion preventing the induction of a pro-inflammatory macrophage phenotype, instead promoting an anti-inflammatory phenotype. This had functional consequences in that macrophages lacking Nr4a1 showed a reduced ability to induce cardiac fibroblast migration. Interestingly, deletion of Nr4a1 in isolated cardiac fibroblasts also had profound effects on their phenotype and function, with these cells not able to produce excess extracellular matrix proteins, convert to a myofibroblast phenotype, or respond to macrophage stimuli. Nr4a1 causes cardiac fibrosis and subsequent diastolic dysfunction by inducing a pro-inflammatory phenotype in macrophages and by pushing cardiac fibroblasts towards a pro-fibrotic phenotype in response to pro-fibrotic stimuli. Nr4a1 is also critical for macrophage/fibroblast interactions.

**Supplementary Information:**

The online version contains supplementary material available at 10.1007/s00018-024-05513-8.

## Introduction

Nr4a1 is an orphan nuclear receptor that belongs to the steroid/thyroid receptor family and has complex biological functions and no known endogenous ligand [1]. Nr4a1 is involved in a wide range of functions related to maintaining homeostasis, and thus, its dysregulation has been implicated in a number of pathological conditions including cancer, metabolic disease, inflammation, and cardiovascular disease [2]. In atherosclerosis, Nr4a1 is protective as it impairs atherosclerotic plaque formation by opposing smooth muscle and endothelial cell proliferation as well as formation of macrophage-derived foam cells [3]. In the heart, Nr4a1 expression is induced by stimuli that cause adverse cardiac remodeling [4, 5], and under conditions of myocardial ischemia Nr4a1 is again protective since its deletion in mice caused a malformed infarct scar, and consequently, reduced cardiac function [6]. While the contribution of Nr4a1 to extra cellular matrix (ECM) production in the infarct zone is important in ischemia where a well-formed scar is essential to maintaining long-term cardiac function, in adverse cardiac remodeling of non-ischemic etiologies (e.g. hypertension, diabetes), excess ECM is detrimental and contributes to diastolic dysfunction [7, 8]. Diastolic dysfunction is an underlying cause of heart failure with preserved ejection fraction that makes up roughly 50% of heart failure cases [9]. Most studies that have investigated Nr4a1 in non-ischemic adverse cardiac remodeling have focused heavily on cardiomyocyte hypertrophy [5, 10] rather than fibrosis, however, deletion of Nr4a1 has been shown to reduce cardiac fibrosis [10, 11].

Multiple cell types contribute to cardiac fibrosis, including macrophages that play an important role in fibrosis in both ischemic and non-ischemic settings [12, 13]. However, the importance of Nr4a1 to macrophage phenotype has only been studied in detail in myocardial ischemia, where Nr4a1 deficiency impaired the resolution of inflammation leading to defective scar formation [6]. The importance of Nr4a1 to macrophage phenotype and function in non-ischemic settings is unknown. Similarly, while Nr4a1 has been shown to regulate important genes related to cardiac fibroblast phenotype and function, the extent to which Nr4a1 contributes to actual cardiac fibroblast phenotype and function, as well as to macrophage-fibroblast interactions is unclear. Accordingly, we used the angiotensin II (Ang II)-infused mouse model of non-ischemic cardiac fibrosis, as well as in vitro cell models, to determine the contribution of Nr4a1 to: (1) cardiac fibrosis and diastolic dysfunction; (2) macrophage phenotype and function; (3) macrophage-fibroblast interactions; and (4) cardiac fibroblast phenotype and function. Together, these approaches identified an important role for Nr4a1 in the development of cardiac fibrosis and diastolic dysfunction via modulation of macrophage and cardiac fibroblast phenotype, including a critical role for Nr4a1 in regulating communication between macrophages and fibroblasts.

## Materials and methods

### Experimental design

Experiments were performed using 8-week-old male wild type (WT) C57BL/6 J and Nr4a1^−/−^ (B6;129S2-*Nr4a1*^*tm1Jmi*^/J) mice. All mice were housed under standard environmental conditions and maintained on standard commercial mouse chow and tap water ad libitum. All procedures were conducted according to the animal ethics protocols RESP/17/203 and 2203051911 approved by the Northern Sydney Local Health District Animal Ethics Committee and West Virginia University Institutional Animal Care and Use Committee, respectively. These studies conformed to the principles of the National Institutes of Health *Guide for the Care and Use of Laboratory Animals*. The Ang II-infusion model was chosen to assess the contribution of Nr4a1 to adverse cardiac remodeling. Mice were randomly divided into four groups: (1) WT + Saline (n = 14); (2) WT + Ang II (1500 ng/kg/min, n = 15); (3) Nr4a1^−/−^ + Saline (n = 13); and (4) Nr4a1^−/−^ + Ang II (n = 13). Ang II and saline were delivered continuously via osmotic minipump (Alzet). For minipump implantation, mice were anaesthetized using inhaled isoflurane (2%). Once anaesthetized, as evaluated by toe pinch reflex, a longitudinal incision was made along the midline of the abdomen and Alzet 3 or 7 day mini-pumps containing either saline or Ang II were peritoneally implanted. 3 day treatment was chosen because it represents early inflammation and 7 day treatment was chosen as fibrosis has developed at this time in the Ang II model. The peritoneum was closed using 5-0 Ethicon chromic gut sutures, while the skin was closed with surgical staples. All mice received Temgesic (0.01 mg/kg) for pain relief. The experimental period lasted either 3 or 7 days as specified. At the experimental endpoint the mice were anaesthetized using isoflurane (2%) and euthanized by removal of the heart. The heart was separated into the left ventricle (LV) plus septum and the right ventricle (RV). The lungs, LV, and RV were weighed, and tibia length measured. The apex portion of the LV was snap frozen in liquid nitrogen and stored at − 80 °C, while the base was fixed in zinc formalin for histology.

### Echocardiography

Mice underwent echocardiographic assessment of LV structure and function (Vevo 3100 Imaging System) at day 0 and at the end of the 7 day experimental period. Mice were anaesthetized with isoflurane (2%) for the procedure. Images were taken at mid-papillary level with a 30 MHz transducer. Measurements in diastole of LV posterior wall thickness (LVPWd) and internal chamber diameter (LVIDd) were made using two-dimensional M-Mode in the parasternal short axis view. LV function was assessed by fractional shortening (FS), stroke volume, and cardiac output calculated as follows:

FS = (LVIDd − LVIDs/LVIDd) × 100,

Stroke volume = LVIDd − LVIDs,

Cardiac Output = stroke volume × heart rate,

where LVIDd and LVIDs represent LV internal diameter in diastole and systole, respectively.

### Left ventricular pressure analysis

At the experimental end-point (day 7), measurements of LV end diastolic pressure (LVEDP), LV end systolic pressure (LVESP), developed pressure, and rates of contraction (dP/d*t*) and relaxation (-dP/d*t*) were made. Briefly, mice were anaesthetized with isoflurane (2%) and placed on a small animal ventilator. The chest cavity was then opened and a high-fidelity Millar conductance catheter was inserted into the LV chamber through the apex of the heart to obtain consistent pressure tracings. Any hearts for which a consistent pressure trace could not be obtained were excluded from the analysis.

### Histology

Picrosirius red-staining of fixed LV sections of 5 μm thickness at the mid papillary level were used to identify fibrillar collagen for determination of collagen volume fraction and cardiac fibrosis. Following rehydration with decreasing concentrations of ethanol, LV sections were stained with picrosirius red (0.1% Sirius Red F3BA in picric acid). The LV sections were then dehydrated using increasing concentrations of ethanol and xylene before being mounted with Depex and cover slipped. Stained LV sections were imaged using a light microscope at 200 × magnification. Ten images per LV section were acquired and each image was analyzed using the Threshold tool in Image J software by highlighting stained collagen fibers. The percentage highlighted area of collagen was measured for each image with the average of the 10 images taken to generate the overall collagen volume fraction for that LV section. Perivascular areas were excluded from the analysis. One section per LV tissue was analyzed with eleven to fifteen LV tissues were used per group in the analysis.

### Immunolabeling

LV sections of 5 μm thickness at the mid papillary level underwent rehydration before incubation in boiling citrate buffer (pH 6) for 60 min for antigen retrieval. The sections were then blocked for non-specific binding before being labelled with antibodies (Table 1). All sections were stained with DAPI solution (Sigma-Aldrich) for 15 min before being cover slipped using VectaShield mounting medium (Vector) and visualized using an EVOS M5000 fluorescent microscope. Macrophage numbers were calculated as average number of positive labeled cells from five fields per LV section at 200 × magnification. One section per LV tissue was analyzed with seven to fifteen LV tissues per group used in the analysis. α-smooth muscle actin (α-SMA) positive cells were quantified across the LV section, excluding the perivascular area and smooth muscle cells. Representative images were taken using the 200 × magnification.

**Table 1:** Antibodies used in LV immunolabeling

	Primary antibody	Dilution	Secondary antibody	Dilution
Mac2 macrophage	Rat anti-Mouse Mac2 (Cedarlane)	1:200 (1 h)	AlexaFluor Goat anti-Rat 568 (Life Technologies)	1:100 (1 h)
CD86 macrophage	Mouse Monoclonal B7-2 (D-6) (Santa Cruz Biotechnology)	1:100 (overnight)	AlexaFluor Goat anti-Mouse 488 (Life Technologies)	1:100 (1 h)
CD206 macrophage	Rabbit anti-Mouse Mannose Receptor (Abcam)	1:100 (overnight)	AlexaFluor Goat anti-Rabbit 647 (Life Technologies)	1:100 (1 h)
α-SMA myofibroblast	Rabbit anti-Mouse α-SMA (Abcam)	1:100 (overnight)	AlexaFluor Goat anti-Rabbit 488 (Life Technologies)	1:100 (1 h)

### Isolation and treatment of cardiac fibroblasts

Cardiac fibroblasts were isolated from the LV of male WT and Nr4a1^−/−^ mice (8 weeks of age, n = 6) similarly to as we have described previously with one preparation per mouse [14–21]. Briefly, LV tissues were isolated, minced and then digested by a series of 5 incubations with 100 ng/µL Liberase TM (Roche) at 37 °C for 15 min with agitation. The cell pellets were resuspended and plated in Dulbecco’s Modified Eagle’s Medium (DMEM)-F12 supplemented with 10% Fetal Bovine Serum (FBS) and 1% Penicillin/Streptomycin (P/S). Non-adherent cells were removed by washing with Mosconas solution. In order to minimize changes in phenotype associated with passaging, fibroblasts were passaged only one time. That is, as they were seeded on 0.1% gelatin-coated 6-well plates in preparation for treatment. Once adhered to the 6-well plates, the fibroblasts were serum-starved in DMEM-F12 for 24 h. Treatments with Ang II (300 nM) and TGF-β1 (30 ng/mL) were then performed in DMEM-F12 containing 1.5% FBS for 24 h. In order to specifically activate Nr4a1, a subset of fibroblasts were treated with the Nr4a1 agonist, Cytosporone B (1 μM), with DMSO as vehicle control also in DMEM-F12 containing 1.5% FBS for 24 h.

### Bone marrow-derived macrophages

Bone marrow cells were extracted from the tibias and femurs of 6 to 8-week-old male WT and Nr4a1^−/−^ mice and centrifuged at 800 rpm for 8 min in DMEM supplemented with 10% FBS and 1% P/S. The pellet was then resuspended and plated in DMEM containing 10 ng/mL of colony stimulating factor (Sigma-Aldrich) for population expansion. 90% pure populations of bone marrow-derived macrophages (BMMΦ) were obtained after 7 days. For in vitro experiments, BMMΦ’s were treated with lipopolysaccharide (LPS, 20 ng/mL) for 6 h or 24 h as specified.

### Cell proliferation and migration

Cell proliferation was determined using the CyQuant NF Proliferation Assay Kit (Life Technologies), according to the manufacturer’s protocol. WT and Nr4a1^−/−^ BMMΦ’s or isolated cardiac fibroblasts were cultured in 96 well plates at 1 × 10^4^ cells/well and treated with Ang II (300 nM) or LPS (20 ng/mL) for 6 h. The assay was performed in duplicate and read using a fluorescence microplate reader at 485 nm wavelength.

Cell migration was assessed using transwell membranes. Briefly, WT or Nr4a1^−/−^ BMMΦ’s were seeded on 12 well plates at 5 × 10^4^ cell density. Then WT or Nr4a1^−/−^ cardiac fibroblasts were seeded on the 8 μm transwell membrane inserts at 2 × 10^4^ cells and placed into each well to obtain the following groups (lower/upper): (1) WT BMMΦ’s/WT fibroblasts, (2) Nr4a1^−/−^ BMMΦ’s/WT fibroblasts; (3) WT BMMΦ’s/Nr4a1^−/−^ fibroblasts; and (4) Nr4a1^−/−^ BMMΦ’s/ Nr4a1^−/−^ fibroblasts. BMMΦ’s were treated with either Ang II (300 nM) or LPS (20 ng/mL) and fibroblasts allowed to migrate through the porous upper level membrane for 24 h. At the end of 24 h, the membranes were fixed in methanol, cut and mounted with Prolong Diamond mounting medium containing DAPI stain (Life Technologies) to identify the migrated fibroblasts, which were then counted under a fluorescent microscope.

### Cell and tissue protein extraction and quantification

LV protein was extracted using tissue specific T-PER lysis buffer containing protease and phosphatase inhibitors (Thermo Scientific). The LV tissues were homogenized in ice using a tissue homogenizer until no tissue pieces were visible. The homogenized tissues were then centrifuged at 10,000 rpm at 4 °C for 10 min and the supernatant collected. For cells, each treatment was collected in separate tubes containing RIPA lysis buffer with protease and phosphatase inhibitors (Thermo Scientific). The cells were then sonicated in an ice bath before being centrifuged at 10,000 rpm at 4 °C for 10 min and the supernatant collected. Proteins from both cell and tissue lysates were then quantified using bicinchoninic acid (BCA) assay according to the manufacturer’s protocol (Thermo Scientific).

### Biochemical assays

A multiplex assay (Biorad) was used to assess the release of TNF-α, IL-1β, IL-2, IL-4, IL-5, IL-6, IL-10, GM-CSF, and CCL2 into the cell culture media by BMMΦ’s in response to LPS (20 ng/mL). Commercial ELISA’s (BD Biosciences) were also used to measure release of TNF-α and IL-6 into the cell culture media from isolated cardiac fibroblasts in response to Ang II (300 nM) and TGF-β1 (30 ng/mL). Commercial ELISA kits were used to measure released collagen Iα1 (Novus Biologicals), fibronectin (MyBioSource), laminin (Abcam) and lysyl oxidase (LOX, MyBioSource) from isolated cardiac fibroblasts. α-SMA (Novus Biologicals), a marker of myofibroblast conversion, was assessed in cell lysates. Commercial ELISA’s were used to measure CCL2 (BD Biosciences) and CCR2 (Aviva Systems Biology) in LV protein extracts. All assays were performed in duplicate from the same samples for both cells, media and LV tissues.

### Real time polymerase chain reaction

Cardiac fibroblasts were isolated from the LV of male WT (8 weeks of age) mice, plated and treated according to the isolation process described above. The cells were then treated with TGF-β1 (30 ng/mL) for 2, 3, 6 and 24 h. RNA was extracted using an RNA isolation kit (Qiagen) according to the manufacturer protocol, and cDNA was synthesized using a SuperScript VILO IV cDNA synthesis kit (Life Technologies). The mRNA levels of *Nr4a1* were quantified using TaqMan Fast Advanced Master Mix (Life Technologies) with the mouse *Nr4a1* gene-specific Taqman probe (Life Technologies) and normalized against mouse *Gapdh* (Taqman probe, Life Technologies). The samples were amplified in duplicate on a QuantStudio3 real time PCR machine (Life Technologies) and the relative gene expression levels (Ct values) were generated by ExpressionSuite Software (Life Technologies).

### RNA-sequencing

Cardiac fibroblasts were isolated from the LV of 7 days Ang II treated male WT and Nr4a1^−/−^ mice (8 weeks of age) according to the isolation process described above. The next day, the non-adherent cells were removed through washing and the adherent cardiac fibroblasts were collected through trypsinization process and washed with sterile PBS. The cells were then spun at 800 g at 4 °C for 10 min. The cell pellets were directly snap-frozen in liquid nitrogen and stored in − 80 °C. RNA extraction, cDNA library generation and 150 bp paired-end Illumina sequencing was performed by before Active Motif, Inc.

The STAR (Spliced Transcripts Alignment to a Reference) v2.7.11a aligner [22] was used to map reads to the *Mus musculus* genome assembly GRCm38/mm10. Fragment counting within annotated features was carried out using HTSeq count 2.0 [23]. Normalization, visualization, and differential expression analysis were conducted using DESeq2 v1.40.2 [24]. Genes with a fold-change greater than 1.2 and a false discovery rate (FDR) less than 0.01 were identified as differentially expressed. Gene set enrichment analysis (GSEA) [25] and visualization of enrichment results was performed using the clusterProfiler v4.6.0 [26, 27]. Gene sets were sourced from the Gene Ontology (GO) database (geneontology.org).

### Statistical analysis

All grouped data were expressed as mean ± SD or SEM as appropriate. Grouped data comparisons were made by t-test, or one-way ANOVA for comparison of three or more groups. When ANOVA analysis identified a significant overall effect, intergroup comparisons were made using the Tukey post-test. Statistical significance was taken at p < 0.05.

## Results

### Nr4a1 deletion reduced Ang II-induced cardiac fibrosis and myofibroblast phenotype conversion

**Fig. 1:**
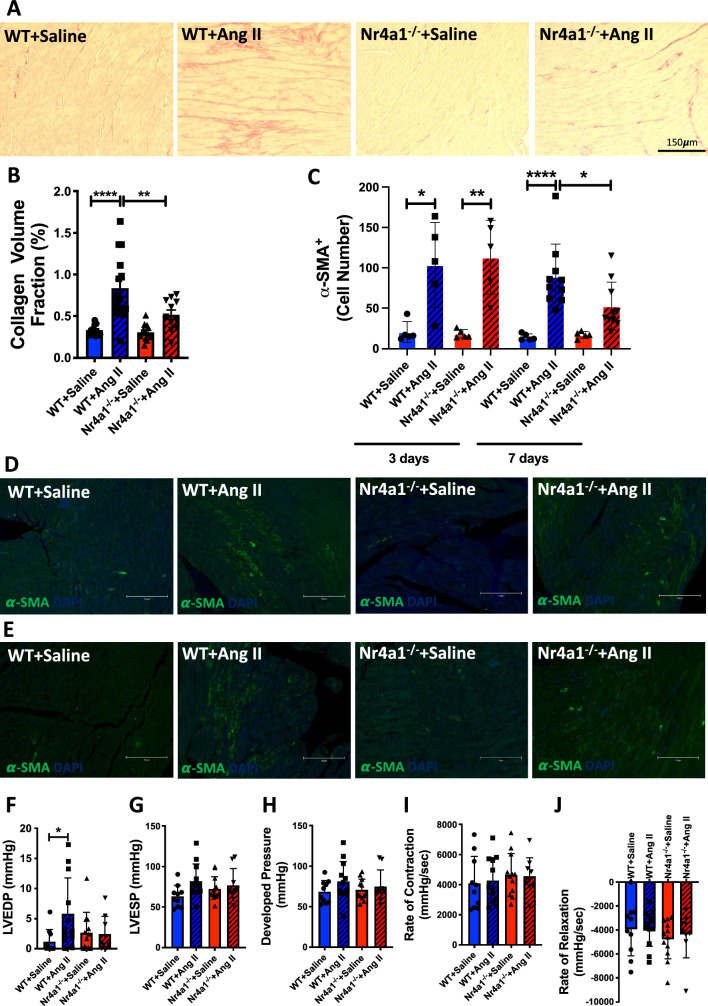
Nr4a1 loss of function reduces cardiac fibrosis, myofibroblast phenotype conversion and improves diastolic function. **A** Representative images of picrosirius red staining of fibrillar collagen for WT + Saline (n = 14), WT + Ang II (n = 15), Nr4a1^−/−^ + Saline (n = 13), and Nr4a1^−/−^ + Ang II (n = 11); and **B** quantification of collagen volume fraction following 7 days of Ang II infusion; **C** quantification of α-SMA myofibroblasts following 3&nbsp;days of Ang II infusion for WT + Saline (n = 5), WT + Ang II (n = 5), Nr4a1−/− + Saline (n = 5), and Nr4a1−/− + Ang II (n = 5) and 7&nbsp;days of Ang II infusion for WT + Saline (n = 5), WT + Ang II (n = 10), Nr4a1−/− + Saline (n = 5), and Nr4a1−/− + Ang II (n = 10). Data are mean ± SD, * = p &lt; 0.05, ** = p &lt; 0.01, *** = p &lt; 0.001, **** = p &lt; 0.0001; **D** representative images of α-SMA^+^ myofibroblasts following 3 days of Ang II infusion; **E** representative images of α-SMA^+^ myofibroblasts following 7 days of Ang II infusion; **F** left ventricular end diastolic pressure (LVEDP), **G** left ventricular end systolic pressure (LVESP), **H** developed pressure, **I** rate of contraction, and **J** rate of relaxation of WT + saline (n = 10), WT + Ang II (n = 12), Nr4a1^−/−^ + saline (n = 12), and Nr4a1^−/−^ + Ang II (n = 9) following 7 days of infusion. Data are mean ± SEM, * = p < 0.05, ** = p < 0.01, **** = p < 0.0001

To confirm that Nr4a1 contributes to Ang II-induced cardiac fibrosis, we implanted WT and Nr4a1^−/−^ mice with either saline or Ang II-filled osmotic mini pumps for 7 days. Whilst Ang II-infused WT mice showed significant cardiac fibrosis as indicated by increased collagen volume fraction (p < 0.0001 versus WT + Saline), Ang II-infused Nr4a1^−/−^ mice did not develop cardiac fibrosis (p < 0.01 versus WT + Ang II, p = 0.10 versus Nr4a1^−/−^ + Saline, Fig. 1A, B). In WT and Nr4a1^−/−^ hearts, Ang II infusion for 3 days increased α-SMA^+^ cells as a marker of myofibroblast phenotype compared to saline treatment (p < 0.05 versus WT + Saline, p < 0.01 versus Nr4a1^−/−^ + Saline, Fig. 1C, D). Whilst the α-SMA^+^ cells remained similar in the wild type hearts at 7 days of Ang II infusion, α-SMA^+^ cells in the Nr4a1^−/−^ hearts were significantly reduced (p < 0.0001 versus WT + Saline, p < 0.01 versus WT + Ang II, Fig. 1C, E). Concomitant with fibrosis, there was a significant increase in LVEDP in Ang II-infused WT mice (p < 0.05 versus WT + Saline, Fig. 1F) that did not occur in Ang II mice lacking Nr4a1 (p = 0.99 versus Nr4a1^−/−^ + Saline). No significant changes were observed between any of the groups for LVESP, developed pressure, rate of contraction or rate of relaxation (Fig. 1G–J).

### Nr4a1 deletion prevents concentric hypertrophy and improves cardiac output

**Fig. 2:**
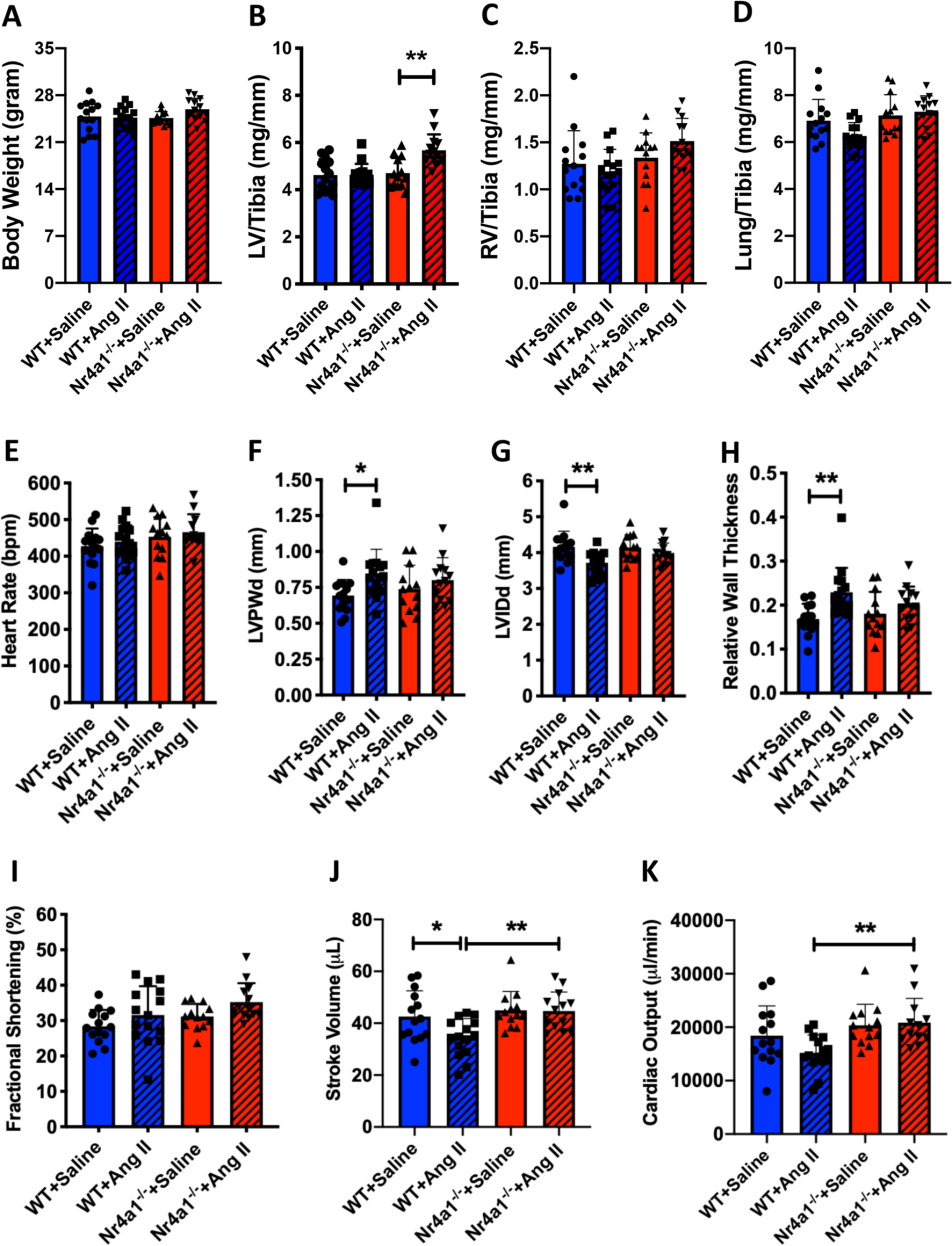
Mouse biometrics and echocardiography. **A** Body weight; **B**–**D** left ventricle, right ventricle and lung weight normalized to tibia length for WT + Saline (n = 14), WT + Ang II (n = 15), Nr4a1^−/−^ + Saline (n = 13), and Nr4a1^−/−^ + Ang II (n = 13). All data are mean ± SD; **E** heart rate, **F** left ventricular posterior wall thickness in diastole (LVPWd), **G** left ventricular internal diameter in diastole (LVIDd), **H** relative wall thickness, **I** fractional shortening, **J** stroke volume, and **K** cardiac output for WT + Saline (n = 14), WT + Ang II (n = 15), Nr4a1^−/−^ + Saline (n = 13), and Nr4a1^−/−^ + Ang II (n = 13) at day 7 post-Ang II. All data are mean ± SEM, * = p < 0.05, ** = p < 0.01

There was no significant difference in body weight between any of the groups at the end of the 7-day experimental period (Fig. 2A). While Ang II did not result in a statistically significant increase in LV/tibia ratio in WT mice, it did cause LV hypertrophy in Nr4a1^−/−^ mice (p < 0.01 versus Nr4a1^−/−^ + Saline, Fig. 2B). No differences in the ratio of RV and lung weight to tibia length were observed between any of the groups (Fig. 2C, D). Echocardiography was used to further assess LV structure and function. There were no differences at baseline between any groups for heart rate, LVPWd, LVIDd, relative wall thickness, or FS (Fig. S1). However, Nr4a1^−/−^ + Ang II mice did have increase stroke volume and cardiac output compared to WT + Ang II at baseline (p < 0.01 for each parameter, Fig. S1). At the end of the 7-day experimental period, heart rate was unchanged between groups (Fig. 2E). LVPWd was increased (p < 0.05 versus WT + Saline, Fig. 2F) and LVIDd decreased (p < 0.01 versus WT + Saline, Fig. 2G) in WT Ang II mice, consistent with concentric hypertrophy and resulting in an increased relative wall thickness (p < 0.01 versus WT + Saline, Fig. 2H). There were no statistically significant increases in these parameters for Nr4a1^−/−^ + Ang II mice. There were no differences in fractional shortening between any groups (Fig. 2I). Stroke volume was reduced in WT + Ang II mice (p < 0.05 versus WT + Saline, Fig. 2J), whereas stroke volume was maintained in Nr4a1^−/−^ + Ang II mice resulting in a significant difference between the two Ang II groups (p < 0.01). While not significantly different, cardiac output tended to be reduced in WT + Ang II mice leading to cardiac output in these mice being significantly reduced in comparison to Nr4a1^−/−^ + Ang II mice (p < 0.01, Fig. 2K).

### Nr4a1 deletion alters left ventricular macrophage infiltration in response to Ang II

**Fig. 3:**
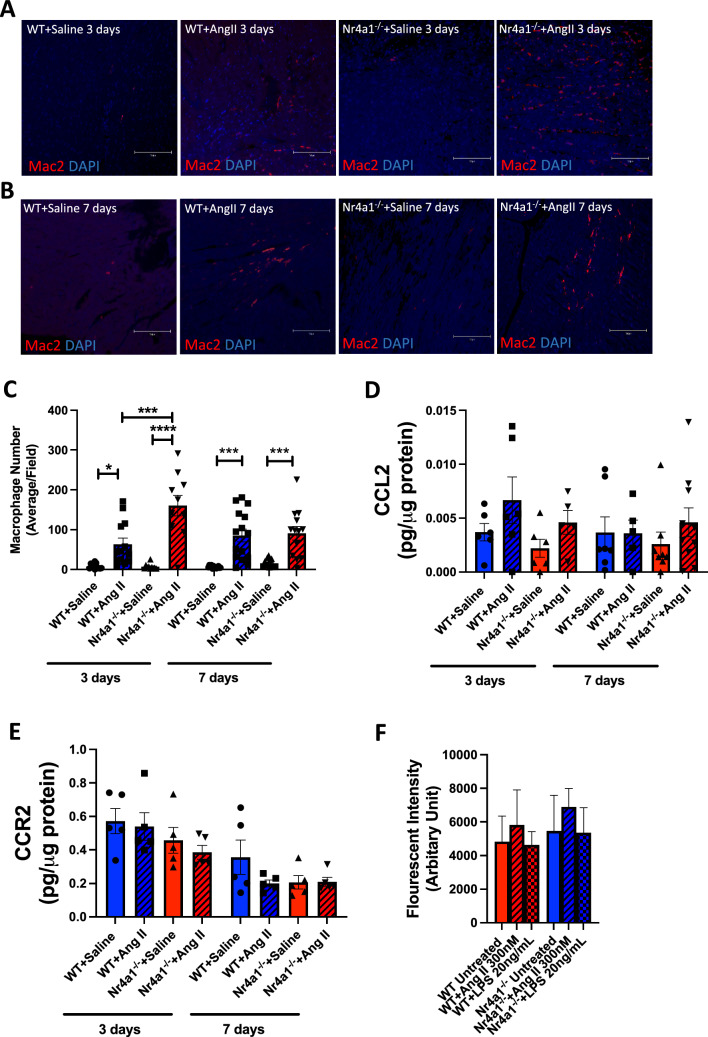
Left ventricular macrophage numbers, CCL2 and CCR2 levels. **A** Representative images of macrophage numbers following 3 days of Ang II infusion; **B** representative images of macrophage numbers following 7 days of Ang II infusion; **C** quantification of macrophage numbers following 3 days and 7 days of Ang II infusion; **D** CCL2 levels following 3 days and 7 days of Ang II infusion; **E** CCR2 levels following 3 days and 7 days of Ang II infusion for WT + Saline (n = 12), WT + Ang II (n = 15), Nr4a1^−/−^ + Saline (n = 13), and Nr4a1^−/−^ + Ang II (n = 11); **F** proliferation of WT and Nr4a1^−/−^ bone marrow derived macrophages treated either with Ang II or LPS. Data are mean ± SEM, * = p < 0.05, *** = p < 0.001, **** = p < 0.0001

Macrophage numbers were assessed in the LV following 3 and 7 days of Ang II infusion. At 3 days, Ang II induced a substantial increase in macrophage number in WT mice (p < 0.05 versus WT + Saline, Fig. 3A and C) that was further increased by 7 days (p < 0.001 versus WT + Saline, Fig. 3A and C). Interestingly, there was a greater increase in macrophage number in Nr4a1^−/−^ + Ang II hearts compared to WT + Ang II mice at 3 days (p < 0.001 versus WT + Ang II, p < 0.0001 versus Nr4a1^−/−^ + Saline, Fig. 3A and C). However, by 7 days, the number of macrophages in Nr4a1^−/−^ + Ang II hearts had decreased to a similar level as in WT + Ang II mice. Both Ang II groups were still significantly higher than their own control group (p < 0.001 WT + Ang II versus WT + Saline, p < 0.001 Nr4a1^−/−^ + Ang II versus Nr4a1^−/−^ + Saline, Fig. 3B and C). Next, we examined LV levels of the macrophage chemokine CCL2 and its receptor CCR2. While there were no significant changes observed in CCL2 levels between any groups at either 3 or 7 days of Ang II (Fig. 3D), there was a trend for CCL2 to be increased at 3 days in both Ang II groups compared to their relative controls. This trend remained for the Nr4a1^−/−^ + Ang II group at 7 days. Similarly for CCR2, there were no statistically significant differences between any of the groups at either 3 or 7 days (Fig. 3E). Macrophage proliferation was assessed using BMMΦ’s in response to Ang II and LPS (Fig. 3F). Neither stimulus caused a statistically significant increase in proliferation and deletion of Nr4a1 did not affect the proliferative response.

### The deletion of Nr4a1 alters bone marrow derived macrophage phenotype

**Fig. 4:**
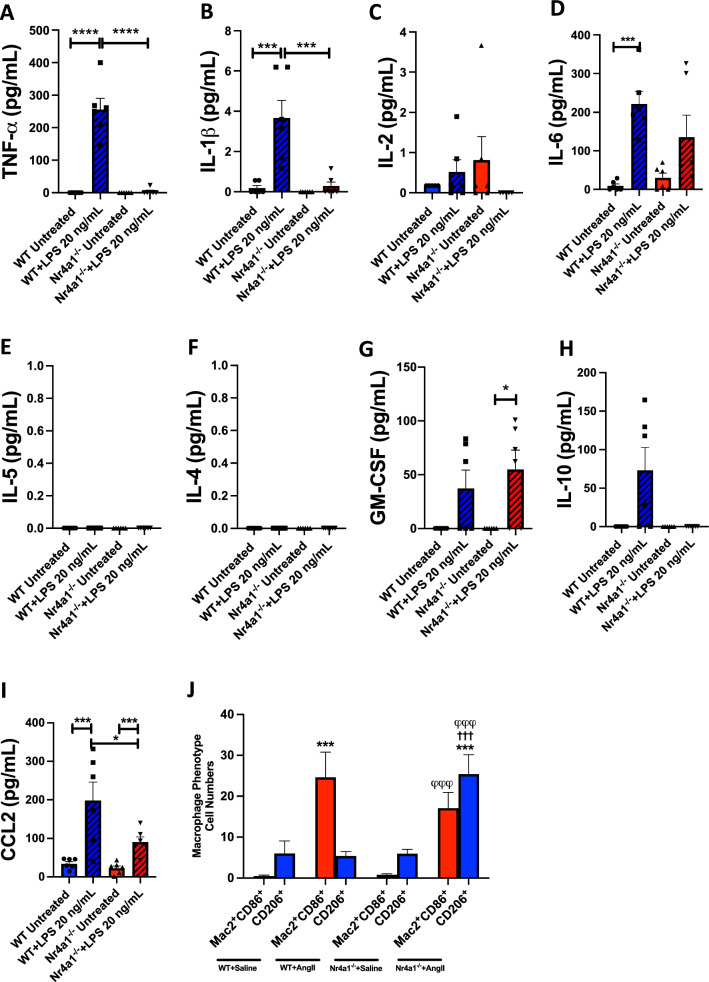
Cytokine release profiles from WT and Nr4a1^−/−^ macrophages and their phenotypes differences in Angiotensin II treated hearts. **A**–**I** TNF-α, IL-1β, IL-2, IL-6, IL-5, IL-4, GM-SCF, IL-10, and CCL2 levels released from WT and Nr4a1^−/−^ macrophages treated with LPS; **J** average cell numbers between pro-inflammatory (Mac2^+^CD86^+^) and anti-inflammatory (CD206^+^) cardiac macrophages in WT + Saline, WT + Ang II, Nr4a1^−/−^ + Saline and Nr4a1^−/−^ + Ang II treated mouse hearts. Data are mean ± SEM, n = 6–7, * = p < 0.05 vs Nr4a1^−/−^ Untreated and WT + LPS, *** = p < 0.001 vs WT Untreated, WT + LPS, WT + Saline, **** = p < 0.0001 vs WT Untreated and WT + LPS, φφφ = p < 0.001 vs Nr4a1^−/−^ + Saline, ††† = p < 0.001 vs WT + Ang II

We utilized BMMΦ’s from WT and Nr4a1^−/−^ mice treated with LPS for proof-of-concept experiments aimed at identifying potential differences in macrophage phenotype. Multiplex assay data presented in Fig. 4A–I revealed that WT macrophages responded to LPS by increasing the release of the inflammatory cytokines TNF-α, IL-1β, IL-6, GM-CSF and CCL2. Nr4a1^−/−^ macrophages did not increase release of TNF-α (p < 0.0001 vs WT LPS) or IL-1β (p < 0.001 vs WT LPS) in response to LPS. Whilst Nr4a1^−/−^ BMMΦ’s did release increased amounts of IL-6 and CCL2, this was less than WT cells for CCL2 (p < 0.05 vs WT LPS). Deletion of Nr4a1 did not alter release of GM-CSF. There was no release of IL-4 and IL-5 from either genotype in response to LPS.

Following these proof-of-concept experiments, we then analyzed macrophage phenotype in the LV of WT and Nr4a1^−/−^ mice infused with Ang II for 7 days as this was the peak macrophage response in the WT mouse. We defined Mac2^+^CD86^+^ cells as pro-inflammatory macrophages and CD206^+^ cells as anti-inflammatory macrophages. Example labelling can be seen in Fig. S2. There were no differences in the numbers of Mac2^+^CD86^+^ and CD206^+^ macrophages between WT and Nr4a1^−/−^ mice receiving saline (Fig. 4J). The number of Mac2^+^CD86^+^ cells was increased in the WT following Ang II (p < 0.001 vs WT + Saline, Fig. 4J). Mac2^+^CD86^+^ cells were also increased in Nr4a1^−/−^ + Ang II mice (p < 0.001 vs Nr4a1^−/−^ + Saline) but to a lesser extent than in WT + Ang II mice (p < 0.05 Nr4a1^−/−^ + Ang II vs WT + Ang II). CD206^+^ cells were unchanged in the WT following Ang II (Fig. 4J), whilst CD206^+^ cells were increased in Nr4a1^−/−^ + Ang II mice (p < 0.001 vs Nr4a1^−/−^ + Ang II, p < 0.001 vs WT + Ang II). We also observed that CD206^+^ cells were increased in Nr4a1^−/−^ + Ang II mice in comparison to the other groups (p < 0.001 vs WT + Saline, WT + Ang II and Nr4a1^−/−^ + Saline, Fig. 4J).

**Fig. 5:**
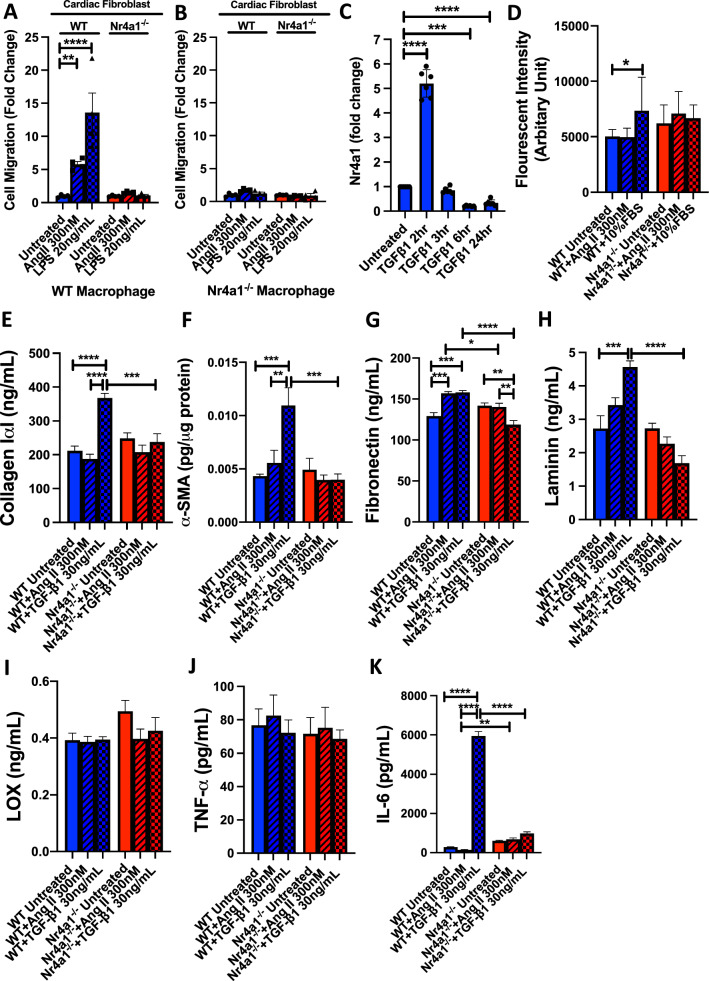
Nr4a1 is essential for a pro-fibrotic cardiac fibroblast phenotype. **A**, **B** Cardiac fibroblast migration in response to macrophage activation with AngII or LPS in the presence or absence of Nr4a1; **C** Nr4a1 transcription level time course in response to TGF-β1; **D** proliferation of WT and Nr4a1^−/−^ cardiac fibroblasts in response to Ang II and 10% fetal bovine serum; **E** collagen Iα1; **F** α-smooth muscle actin (αSMA) in cell lysate; **G** fibronectin; **H** laminin; and **I** lysyl oxidase (LOX) levels in culture media for WT and Nr4a1^−/−^ cardiac fibroblasts treated with Ang II and TGF-β1; **J** TNF-α; **K** IL-6 levels in culture media for WT and Nr4a1^−/−^ cardiac fibroblasts treated with Ang II and TGF-β1. Data are mean ± SEM, n = 6/group, * = p < 0.05, ** = p < 0.01, *** = p < 0.001, **** = p < 0.0001

### Nr4a1 deletion alters macrophage and cardiac fibroblast interactions in vitro

We examined the functional significance of Nr4a1-dependant differences in macrophage phenotype by examining cardiac fibroblast migration in response to macrophage activation. We seeded WT or Nr4a1^−/−^ cardiac fibroblasts on porous 8 μm transwell membranes and placed them inside wells seeded with WT or Nr4a1^−/−^ macrophages treated with either Ang II or LPS. The number of migrated WT cardiac fibroblasts increased significantly in response to Ang II and LPS stimulated WT macrophages (p < 0.01 and p < 0.0001 respectively, Fig. 5A). Conversely, there was no increase in WT cardiac fibroblast migration in response to activated Nr4a1^−/−^ macrophages (Fig. 5B). Nr4a1^−/−^ cardiac fibroblast migration was inhibited in response to Ang II and LPS stimulated WT macrophages (Fig. 5A). There was also no increase in cell migration of both WT and Nr4a1^−/−^ cardiac fibroblasts in a response to either Ang II or LPS stimulated Nr4a1^−/−^ macrophages (Fig. 5B).

### Nr4a1 deletion alters cardiac fibroblast phenotype

Having observed an inability of Nr4a1^−/−^ cardiac fibroblasts to migrate in response macrophage stimuli, we examined the *Nr4a1* mRNA response in cardiac fibroblasts treated with TGF-β1, a potent pro-fibrotic stimulus. We found that *Nr4a1* gene transcription was significantly upregulated in response to TGF-β1 treatment (30 ng/mL) as early as 2 h post-treatment (Fig. 5C). Nr4a1 mRNA levels returned to normal by 3 h and remained there at 6 and 24 h. We then studied the effect of Nr4a1 deletion on cardiac fibroblast phenotype in response to pro-fibrotic stimuli (Ang II and TGF-β1). While 10% serum as a positive control induced a proliferative response by cardiac fibroblasts (p < 0.05), Ang II at 300 nM did not promote proliferation in either WT or Nr4a1^−/−^ cardiac fibroblasts (Fig. 5D). Collagen I release also did not significantly increase in either group in response to Ang II, however, it was significantly increased in WT cardiac fibroblasts in response to TGF-β1 (p < 0.0001 versus WT Untreated, Fig. 5E). In contrast, TGF-β1 did not increase collagen I release by Nr4a1^−/−^ cardiac fibroblasts (p < 0.001 versus WT + TGF-β1, Fig. 5E). In order to corroborate this finding, we also treated WT cardiac fibroblasts with the Nr4a1 agonist, Cytosporone B (1 μM) and measured the release of collagen I. We found that collagen I release was significantly increased in response to Cytosporone B (p < 0.01 versus Untreated and p < 0.05 versus Vehicle) (Fig. S3), confirming Nr4a1 activation induces a pro-fibrotic cardiac fibroblast response. For α-SMA we observed an increase in WT cardiac fibroblasts treated with TGF-β1 (p < 0.001 versus WT Untreated), whilst no increased was observed in Nr4a1^−/−^ fibroblasts treated with TGF-β1 (p < 0.001 versus WT + TGF-β1, Fig. 5F). There was a significant increase in fibronectin release for both WT fibroblasts treated with Ang II or TGF-β1 compared to the untreated WT fibroblasts (p < 0.001 versus WT Untreated, Fig. 5G). However, fibronectin release was not upregulated in fibroblasts lacking Nr4a1 and treated with Ang II or TGF-β1 (p < 0.05 versus WT + Ang II, p < 0.0001 versus WT + TGF-β1, Fig. 5G). Whilst there was a significant increase in laminin release in WT fibroblast treated with TGF-β1 (p < 0.001 versus WT Untreated), there was a profound decrease in laminin levels in Nr4a1^−/−^ fibroblasts treated with TGF-β1 in comparison to the WT counterpart (p < 0.0001 versus WT + TGF-β1, Fig. 5H). For LOX, no significant changes were observed across any treatment groups (Fig. 5I). We also found no difference in TNF-α release across treatment groups for both WT and Nr4a1^−/−^ cardiac fibroblasts (Fig. 5J). There was, however, a significant increase in IL-6 release from TGF-β1 treated WT cardiac fibroblasts (p < 0.0001 versus WT Untreated, Fig. 5K). This increase did not occur in Nr4a1^−/−^ cardiac fibroblasts (p < 0.0001 versus WT + TGF-β1, Fig. 5K).

### Transcriptomic alterations to cardiac fibroblasts lacking Nr4a1

**Fig. 6:**
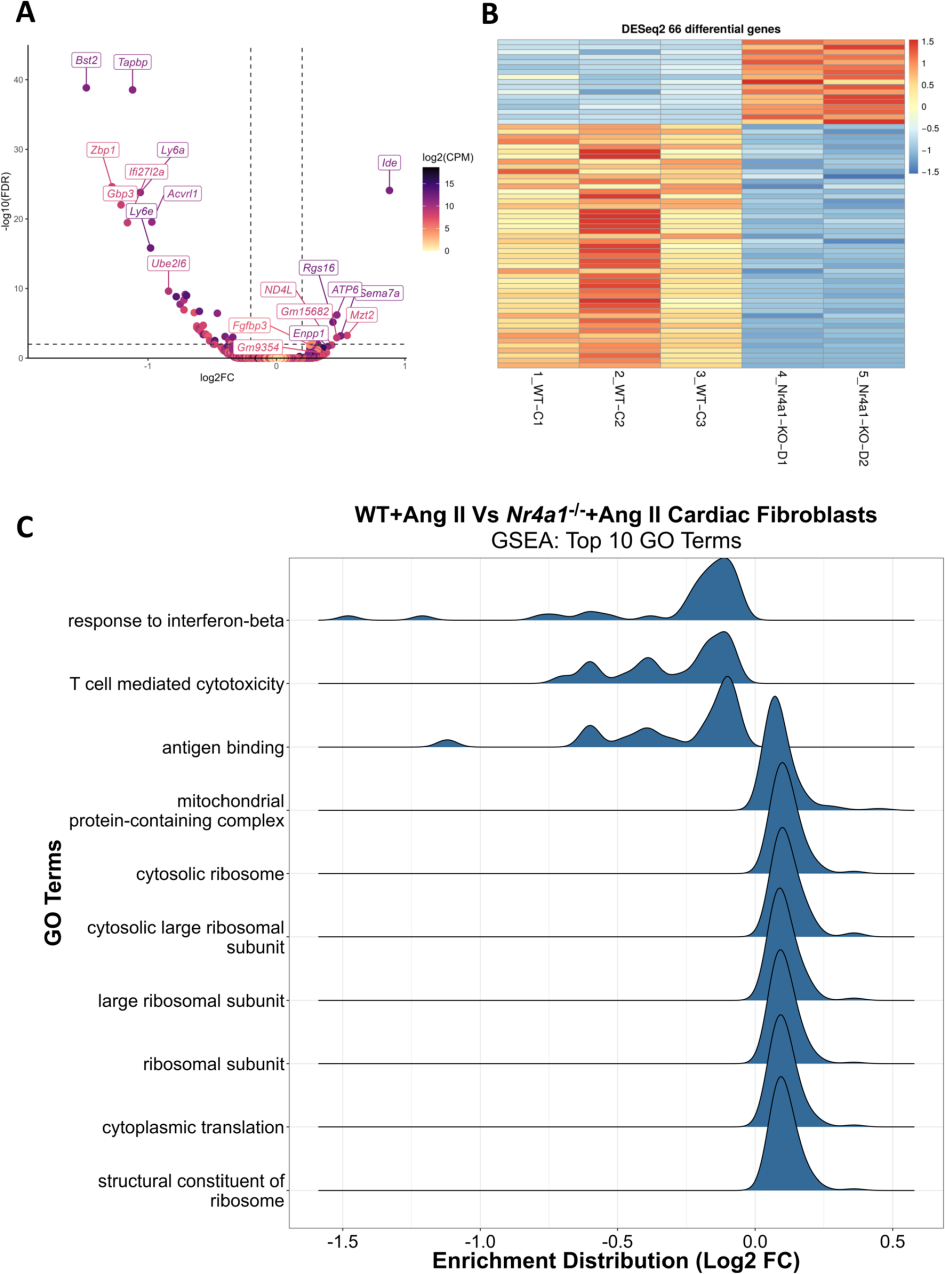
Differentiated gene expression changes in cardiac fibroblasts following Ang II treatment in Nr4a1^−/−^ mice compared to Ang II treated WT mice. **A** Volcano plot; and **B** Heatmap indicating differentially expressed genes between WT + Ang II (n = 3) and Nr4a1^−/−^ + Ang II (n = 2) cardiac fibroblasts. In the volcano plot, genes are colored red when the shrunken log2FC is less than one (log2FC < 1) and green when the shrunken log2FC is greater than one (log2FC > 1); **C** gene set enrichment analysis of the RNA-seq gene set with GO terms. The ridge plot shows the top ten GO terms significantly associated (FDR < 0.01) with the RNA-seq gene set, with ridge shape representing the log2 fold-change distribution of genes enriched within each term

Gene expression changes were determined in cardiac fibroblasts isolated from 7 days of WT + Ang II and Nr4a1^−/−^ + Ang II LVs. RNA-sequencing revealed 66 differentially regulated genes (FDR < 0.01) (Table 2): 17 were up-regulated and 49 were down-regulated in fibroblasts from Nr4a1^−/−^ + Ang II LVs, as illustrated in the volcano plot and heat map (Fig. 6A, B). GSEA showed that the down-regulated genes were associated with responses to IFN-β, T cell mediated toxicity, and antigen binding, while up-regulated genes were linked to mitochondrial function, cytoplasmic translation, and structural constituents of ribosomes in fibroblasts from Nr4a1^−/−^ + Ang II LVs (Fig. 6C).

**Table 2:** Differentially regulated genes in Nra41^−/−^ + Ang II cardiac fibroblasts identified through RNA-sequencing

Gene ID	Gene symbol	Name	Shrunken Log2FC Nr4a1^−/−^/WT	Adjusted p value
19039	Lgals3bp	Lectin galactoside-binding soluble 3 binding protein	– 1.8	9.00E– 89
69550	Bst2	Bone marrow stromal sell antigen 2	– 1.48	1.44E– 39
58203	Zbp1	Z-DNA binding protein 1	– 1.28	2.37E– 25
55932	Gbp3	Guanylate Binding Protein 3	– 1.21	9.62E– 23
76933	Ifi27l2a	Interferon alpha inducible protein 12 like 2a	– 1.16	3.43E– 20
21356	Tapbp	Tap binding protein	– 1.12	2.86E– 39
110454	Ly6a	Lymphocyte antigen 6 family member a	– 1.06	1.58E– 24
17069	Ly6e	Lymphocyte antigen 6 family member e	– 0.98	1.49E– 16
11482	Acvrl1	Activin a receptor like type 1	– 0.97	2.82E– 20
56791	Ube2l6	Ubiquitin conjugating enzyme e2 l6	– 0.84	2.38E– 10
66141	Ifitm3	Interferon-induced transmembrane protein 3	– 0.78	1.48E– 09
16859	Lgals9	Lectin, galactoside-binding soluble 9	– 0.75	1.79E– 08
54123	Irf7	Interferon regulatory factor 7	– 0.72	4.45E– 09
246730	Oas1a	2′−5′ oligoadenylate synthase 1a	– 0.72	1.16E– 07
11603	Agrn	Agrin	– 0.71	7.06E– 10
12010	B2m	Beta-2-microglobulin	– 0.7	1.02E– 09
99899	Ifi44	Interferon induced protein 44	– 0.64	2.93E– 07
327959	Xaf1	XIAP-associated factor 1	– 0.62	2.43E– 05
219132	Phf11d	PHD finger protein 11	– 0.62	3.87E– 05
68708	Rabl2	Rab-like protein 2	– 0.61	6.39E– 05
14972	H2-K1	Histocompatibility 2, K region locus 1	– 0.6	1.88E– 07
15015	H2-Q4	Histocompatibility 2, Q region locus 4	– 0.6	5.04E– 05
15040	H2-T23	Histocompatibility 2, T region locus 23	– 0.6	8.84E– 05
15959	Ifit3	Interferon induced protein with tetratricopeptide repeats 3	– 0.59	0.00013172
100038882	Isg15	Interferon stimulated gene 15	– 0.57	1.94E– 05
17392	Mmp3	Matrix metallopeptidase 3	– 0.57	0.00041476
64685	Nmi	N-myc and STAT interactor	– 0.55	0.00036102
15957	Ifit1	Interferon induced protein with tetratricopeptide repeats 1	– 0.53	0.00070802
23962	Oasl2	2′−5′ oligoadenylate synthase-like 2	– 0.52	0.00332195
170942	Erdr1	Erythroid differentiation Regulator 1	– 0.49	0.00957584
14964	H2-D1	Histocompatibility 2, D region locus 1	– 0.48	0.00299481
16913	Psmb8	Proteasome subunit beta type 8	– 0.48	0.01020777
14862	Gstm1	Glutathione s-transferase mu 1	– 0.46	3.58E– 07
330256	Gm20559	Predicted gene 20559	– 0.43	0.0251338
110557	H2-Q6	Histocompatibility 2, Q region locus 6	– 0.42	0.04591658
53945	Slc40a1	Solute carrier family 40 member 1	– 0.41	0.08120315
66940	Shisa5	Shisha family member 5	– 0.4	0.00079017
80898	Erap1	Endoplasmic reticulum aminopeptidase 1	– 0.4	0.04429441
17294	Mest	Mesoderm specific transcript	– 0.39	0.08223237
11758	Prdx6	Peroxiredoxin 6	– 0.38	0.01957879
19186	Psme1	Proteasome activator subunit 1	– 0.37	0.00264063
12257	Tspo	Translocator protein	– 0.36	0.02206183
20304	Ccl5	C–C motif ligand 5	– 0.35	0.00790233
14962	Cfb	Complement factor b	– 0.35	0.06493054
12258	Serping1	Serpin family G member 1	– 0.34	0.00098468
56615	Mgst1	Microsomal glutathione S-transferase	– 0.34	0.01733032
66168	Grina	Glutamate ionotropic receptor NMDA type subunit associated protein 1	– 0.34	0.06604576
74202	Fblim1	Filamin binding lim protein 1	– 0.27	0.03676885
433375	Creg1	Cellular repressor of e1a stimulated genes 1	– 0.27	0.09859321
212753	Gm4779	Predicted gene 4779	0.26	0.01330106
99889	Arfip1	ADP ribosylation factor interacting protein 1	0.26	0.04434663
382231	8030474K03Rik	8030474K03 Riken	0.28	0.0038602
76477	Pcolce2	Procollagen c-endopeptidase enhancer 2	0.28	0.01668317
17709	COX2	Cyclooxygenase-2	0.32	0.00918231
100039826	NA	Neuroamidase	0.32	0.06604576
107182	Btaf1	B-TFIID TATA-Box binding protein associated factor 1	0.33	0.07815281
67248	Rpl39	Ribosomal protein l39	0.36	0.00013172
16997	Ltbp2	Latent transforming growth factor beta binding protein 2	0.36	0.03318132
18605	Enpp1	Ectonucluotide pyrophosphatase/ phosphodiesterase 1	0.4	0.02166192
17720	ND4L	NADH-ubiquinone oxidoreductase chain 4l	0.43	0.01329532
19734	Rgs16	Regulator of g protein signaling 16	0.44	6.82E– 06
17705	ATP6	Atp synthase membrane subunit 6	0.47	6.28E– 07
100504174	Gm15682	Predicted gene 15682	0.47	0.00114543
20361	Sema7a	Semaphorin 7a	0.5	0.00064113
72083	Mzt2	Mitotic spindle organizing protein 2	0.55	0.00054677
15925	Ide	Insulin degrading enzyme	0.88	8.06E– 25

## Discussion

Nr4a1 has been widely studied in different disease models ranging from cancer [28, 29] to metabolic diseases such as type 2 diabetes [30]. Herein, we utilized the Ang II-infused mouse model of adverse cardiac remodeling, as well as in vitro macrophage and cardiac fibroblast cell models to understand the contribution of Nr4a1 to cardiac fibrosis. We demonstrated that: (1) the absence of Nr4a1 reduced fibrosis and myofibroblast phenotype conversion as well as maintained diastolic function in Ang II-infused mouse hearts; (2) although cardiac macrophage numbers were greater with the absence of Nr4a1, Nr4a1 deletion caused a phenotype shift away from the pro-inflammatory Mac2^+^CD86^+^ phenotype toward the anti-inflammatory CD206^+^ phenotype; (3) this shift in macrophage phenotype alters communication between macrophages and cardiac fibroblasts; and (4) Nr4a1 is required for a pro-fibrotic cardiac fibroblast phenotype.

Ang II-infusion leads to the excess production of ECM proteins characteristic of fibrosis, which manifests as diastolic dysfunction, impaired LV filling and ultimately heart failure [31, 32]. In this study, we report that mice lacking Nr4a1 have significantly reduced cardiac fibrosis in response to 7 days of Ang II. Our finding is in agreement with Wang et al. [10] who also reported that cardiac fibrosis was significantly decreased in Nr4a1^−/−^ mice after four weeks of Ang II, and with Hu et al. [11] who reported reduced fibrosis in a mouse model of doxorubicin-induced cardiotoxicity in Nr4a1^-/-^ mice. In our study, Nr4a1 deletion not only prevented fibrosis, but also maintained normal LVEDP, indicative of prevention of the mild diastolic dysfunction that occurred with Ang II-infusion. Furthermore, while Ang II caused a reduction in LV chamber size and increased wall thickness consistent with concentric hypertrophy, Nr4a1 deletion prevented this concentric remodeling. Systolic function was similar between Ang II groups, however, because stroke volume was reduced in the WT + Ang II group due to the decreased chamber size, cardiac output was subsequently reduced in WT + Ang II mice, but not Nr4a1^−/−^ + Ang II mice. These findings are particularly interesting when compared with ischemia. Whilst the reduction in fibrosis brought about by the absence of Nr4a1 proved to be advantageous in the non-ischemic model used in our study, this is an interesting contrast to myocardial ischemia. Hilgendorf et al. [6] reported a reduction in collagen density in the scar region in Nr4a1^−/−^ mice 21 days post-myocardial ischemia. Consequently, Nr4a1 deletion led to impairment of LV systolic function, likely due to the improper scar formation. Hence Nr4a1 deletion reduces ECM production in both myocardial ischemia and Ang II models, however, while this is advantageous in a non-ischemic setting (i.e. Ang II), it is detrimental in ischemia where proper scar formation is essential to long-term cardiac function.

Following Ang II-infusion, focal cardiomyocyte necrosis results in infiltration of macrophages that in addition to clearing debris also recruit cardiac fibroblasts and promote myofibroblast conversion and ECM deposition. While we found no differences in macrophage numbers between genotypes at baseline, in WT mice, Ang II promoted increased numbers of cardiac macrophages at 3 days, that peaked at 7 days, consistent with other studies [33–35]. However, macrophage numbers in Nr4a1^−/−^ mice peaked at day 3 and were significantly increased compared to WT + Ang II mice at that time-point before becoming similar between Ang II groups by day 7. This enhanced macrophage response in Nr4a1^−/−^ mice is again in agreement with Hilgendorf et al. [6] who also reported increased numbers of monocytes and macrophages in the LV of Nr4a1^−/−^ mice in the days following myocardial ischemia. The increase in macrophages in Nr4a1^−/−^ mice is unlikely to be explained by a higher rate of proliferation since we found that genotype did not affect proliferation of BMMΦ’s, whether untreated or stimulated. We also assessed LV levels of the macrophage recruitment signal CCL2 and its receptor CCR2. There were no statistically significant differences in LV CCR2 levels. LV CCL2 was increased in both WT + Ang II mice and Nr4a1^−/−^ + Ang II mice, although, the relative increase was approximately 81% in WT + Ang II mice compared to 109% for Nr4a1^−/−^ mice following 3 days of Ang II infusion. Thus, there was a roughly 28% greater relative increase in the CCL2 recruitment signal in Nr4a1^−/−^ mice, which may at least partly account for the increase in macrophage numbers. Reduced apoptosis with the absence of Nr4a1 could also be a factor, since in another study, the introduction of a dominant negative mutant Nr4a1 to RAW 264.7 macrophages was able to suppress apoptosis [36], indicating that macrophages lacking effective Nr4a1 live longer.

Since Nr4a1^−/−^ mice had reduced fibrosis despite increased macrophage numbers, this led us to consider that differences in phenotype may exist between WT and Nr4a1^−/−^ macrophages. As proof-of-concept, we treated BMMΦ from WT and Nr4a1^−/−^ mice with LPS and found clear differences in cytokine release. We then used a series of cell-surface markers to identify macrophage phenotype in WT and Nr4a1^−/−^ mouse LV following Ang II-infusion. The take-home message from these experiments is that Ang II increases Mac2^+^CD86^+^ pro-inflammatory macrophages in WT hearts to a greater extent than in Nr4a1^−/−^ hearts, and that anti-inflammatory CD206^+^ macrophages are increased in Nr4a1^−/−^ hearts. Overall, this leads to a more favorable anti-inflammatory environment in Nr4a1^−/−^ hearts, which is consistent with less fibrosis. Although it is not entirely clear if the observed effects are due to changes in macrophage phenotype or simply their increased numbers in Nr4a1^−/−^ mice. Nr4a1-related phenotypic differences in monocytes and macrophages are consistent with previous studies, although the response differs. Splenic monocytes derived from Nr4a1^−/−^ mice showed higher levels of TNF-α compared to WT when stimulated with LPS. Moreover, peritoneal macrophages derived from ApoE^−/−^Nr4a1^−/−^ mice in an atherosclerosis mouse model showed enhanced levels of IL-12, iNOS, and major histocompatibility complex II (MHCII) compared to ApoE^−/−^ peritoneal macrophages, supporting the notion that the absence of Nr4a1 increases macrophage activation in atherosclerosis [3]. However, similar to our study, there was a substantial increase in Ly-6C^low^ macrophages (analogous to anti-inflammatory macrophages) in the Nr4a1^−/−^ heart over the WT heart following ischemia [6]. That study also reported a greater increase in Ly-6C^high^ macrophages (analogous to pro-inflammatory macrophages) in the Nr4a1^−/−^ heart over the WT. Thus, the differences in the contribution of Nr4a1 to macrophage phenotype between organs/tissues indicates that the function of Nr4a1 in regulating inflammatory responses is organ and stimulus specific.

To assess the functional significance of Nr4a1 regulation of macrophage phenotype, we co-cultured macrophages with isolated cardiac fibroblasts in transwell experiments to assess fibroblast migration. Deletion of Nr4a1 in macrophages had the effect of reducing cardiac fibroblast migration, indicating the requirement of Nr4a1 by macrophages to produce molecules that induce a fibroblast response. Of particular interest, we also found that Nr4a1 in fibroblasts was critical to their own ability to respond to a macrophage signal. A previous study reported that siRNA knockdown of Nr4a1 in neonatal cardiac fibroblasts prevented proliferation, migration, and myofibroblast conversion in response to isoproterenol, and also prevented the isoproterenol-induced increase in the genes for α-SMA, periostin, collagen I, and fibronectin [5]. In light of these and our own findings, we sought to further understand Nr4a1 regulation of cardiac fibroblast phenotype by measuring actual release of ECM proteins by adult cardiac fibroblasts isolated from WT and Nr4a1^−/−^ mice. While Ang II had no effect on collagen I production or myofibroblast conversion (α-SMA) in our hands, TGF-β1 induced a robust conversion to the myofibroblast phenotype and increased collagen I production in WT cells; these responses were eliminated by deletion of Nr4a1. Cytosporone B, a natural agonist of Nr4a1 [37], induced a significant release of collagen I, confirming that Nr4a1 is pro-fibrotic in cardiac fibroblasts. Upregulation of collagen I protein induced by Cytosporone B was also observed in human proximal tubular cell line HK-2 cells [38]. Similarly, fibronectin and laminin release that were up-regulated by Ang II and TGF-β1, were decreased in cardiac fibroblasts lacking Nr4a1. Interestingly, these outcomes appear to contrast with the role of Nr4a1 in fibroblasts from some other organs. In a mouse model of skin fibrosis, the loss of Nr4a1 exacerbated fibrosis, shown by pronounced dermal thickening and higher myofibroblast count [39]. In addition, Nr4a1^−/−^ mice are more prone in developing pulmonary fibrosis induced by intra-tracheal instillation of bleomycin [39]. Therefore, the role of Nr4a1 and its regulatory effects in fibroblasts may also be organ specific similar to macrophages.

Effects of Nr4a1 deletion on cardiac fibroblast phenotype were also observed in vivo*,* where myofibroblast numbers in response to 7 days (although not 3 days) of Ang II were reduced by Nr4a1 deletion. Several possibilities may explain this reduction in myofibroblasts: (1) Myofibroblast conversion is inhibited—this is supported by our in vitro fibroblast data. However, if this was a mechanism in vivo, then we would expect myofibroblast numbers to also be reduced in Nr4a1^−/−^ mice at 3 days, especially given that our data indicates that Nr4a1 is an early response gene; (2) Proliferation is inhibited—Nr4a1 deletion did reduce proliferation in response to high serum in vitro, however, again we would expect reduced myofibroblasts in Nr4a1^−/−^ mice at 3 days; (3) This leaves us with increased cell death (apoptosis) resulting in fewer myofibroblasts. Given that reduced cell lifespan may still mean that cells are alive for days, this may explain the lack of difference at 3 days. However, our in vitro proliferation assay labels all cells, thus, one would expect fewer Nr4a1^−/−^ fibroblasts present at baseline in that assay if cell death were increased by Nr4a1 deletion; this was not the case. Thus, the mechanisms underlying reduced myofibroblasts at 7 days are unclear and likely also to be influenced by macrophage phenotype at each time-point given the critical nature of Nr4a1 to macrophage-fibroblast interactions as we discovered. We also found that Nr4a1 deletion prevented the increased release of IL-6 that occurred in WT cardiac fibroblasts in response to TGF-β1, indicating that fibroblast Nr4a1 may also regulate the inflammatory environment. RNA-sequencing of cardiac fibroblasts from the hearts of WT and Nr4a1^−/−^ mice receiving Ang II seems to support this concept with GO terms related to inflammation and immune cell function featuring prominently in the genes down-regulated in Nr4a1^−/−^ fibroblasts. It is important to note though, that because these fibroblasts were isolated from the heart following Ang II and not treated in vitro, some or all genes regulated could be the indirect result of the altered phenotype of macrophages in the heart due to Nr4a1 deletion rather than direct effects of Nr4a1 in the fibroblast itself.

This study has several limitations. Firstly, while we have identified the critical nature of Nr4a1 to macrophage/fibroblast interactions, we have not explored the exact mechanisms underlying this interaction. Secondly, there are likely compensatory mechanisms that occur in response to Nr4a1 deletion. However, regardless of this, Nr4a1 deletion has clear effects and Nr4a1 is clearly involved in fibrosis in the heart. Thirdly, our study investigates two time-points. One of these corresponds to early inflammation and the other to fibrosis. These are the key outcomes of this study. However, the cardiac remodelling process is dynamic, and these two time-points may not capture the full spectrum of Nr4a1 involvement in cardiac remodelling.

In summary, Nr4a1 plays a pro-fibrotic role in Ang II infused mice, leading to mild diastolic dysfunction. Nr4a1 is critical to induction of a pro-inflammatory macrophage phenotype, a pro-fibrotic cardiac fibroblast phenotype, as well as macrophage/fibroblast interactions.

## Supplementary Information

Below is the link to the electronic supplementary material.

Supplementary file1 (DOCX 6120 KB)

## Data Availability

Supporting data for this manuscript are available in the Supplementary Material and all other data are available upon request.
