# Supplementary material for: The orphan nuclear receptor Nr4a1 contributes to interstitial cardiac fibrosis via modulation of cardiac fibroblast and macrophage phenotype
Source: Cell Mol Life Sci. 2024 Dec 7;81(1):484. doi: 10.1007/s00018-024-05513-8 (PMC11625040; doi:10.1007/s00018-024-05513-8)
Supplement: Supplementary file 1 — Supplementary file1 (DOCX 6120 KB) [file 18_2024_5513_MOESM1_ESM.docx]

Supplementary Material

The Orphan Nuclear Receptor Nr4a1 Contributes to Interstitial Cardiac Fibrosis via Modulation of Cardiac Fibroblast and Macrophage Phenotype

^1,2,3^Alexander Widiapradja, PhD, ^1^Heather Connery, BS, ^2^Ainsley O. Kasparian, BS, ^2,3,4^Martyn Bullock, PhD, ^2,3,4^Roderick Clifton-Bligh, MBBS, ^1,2,3^Scott P. Levick, PhD

^1^Department of Physiology, Pharmacology, and Toxicology, West Virginia University Health Sciences Center, Morgantown, WV, USA

^2^Kolling Institute, St Leonards, NSW, Australia

^3^The University of Sydney, Camperdown, NSW, Australia

^4^Royal North Shore Hospital, St Leonards, NSW, Australia

**Corresponding Author:**

Alexander Widiapradja, Ph.D

Department of Physiology, Pharmacology, and Toxicology

West Virginia University Health Sciences Center

Morgantown, WV 26506

Telephone: +1 304-293-0688

Email: alexander.widiapradja@hsc.wvu.edu

**Supplementary Figure 1.** **Mouse echocardiography at baseline. (A)** Heart rate, **(B)** Left ventricular posterior wall thickness in diastole (LVPWd), **(C)** Left ventricular internal diameter in diastole (LVIDd), **(D)** Relative wall thickness, **(E)** Fractional shortening, **(F)** Stroke volume, and **(G)** Cardiac output for WT+Saline (n=14), WT+Ang II (n=15), Nr4a1^-/-^+Saline (n=13), and Nr4a1^-/-^+Ang II (n=13) at baseline. All data are ± SEM, *=p<0.05, **=p<0.01.

**Supplementary Figure 2.** **Macrophage phenotypes in WT and Nr4a1^-/-^ mice treated with Ang II.** Representative images of macrophage phenotypes following 7 days of either Saline or Ang II infusion. Pro-inflammatory macrophage phenotype is represented by double labelling of CD86 (green) and Mac2 (red). Anti-inflammatory macrophage phenotype is represented by CD206 (magenta). Nuclear staining is represented as DAPI (blue). Images were taken at 200x magnification.

**Supplementary Figure 3. Nr4a1 agonist induces a profibrotic response in mouse cardiac fibroblasts.** Activation of Nr4a1 by Cytosporone B significantly increases collagen I release. All data are mean ± SEM, n= 6, *=p<0.05 vs Vehicle, **=p<0.01 vs Untreated.
